# HERG1A potassium channel is the predominant isoform in head and neck squamous cell carcinomas: evidence for regulation by epigenetic mechanisms

**DOI:** 10.1038/srep19666

**Published:** 2016-01-20

**Authors:** Sofía T. Menéndez, M. Ángeles Villaronga, Juan P. Rodrigo, Saúl Álvarez-Teijeiro, Rocío G. Urdinguio, Mario F. Fraga, Carlos Suárez, Juana M. García-Pedrero

**Affiliations:** 1 Servicio de Otorrinolaringología, Hospital Universitario Central de Asturias and Instituto Universitario de Oncología del Principado de Asturias, Oviedo, Spain; 2 Unidad de Epigenética del Cáncer, Instituto Universitario de Oncología del Principado de Asturias, Universidad de Oviedo, Spain; 3 Department of Immunology and Oncology, National Center for Biotechnology, CNB-CSIC, Cantoblanco, Madrid E-28049, Spain

## Abstract

Evidences indicate that HERG1 voltage-gated potassium channel is frequently aberrantly expressed in various cancers including head and neck squamous cell carcinomas (HNSCC), representing a clinically and biologically relevant feature during disease progression and a potential therapeutic target. The present study further and significantly extends these data investigating for the first time the expression and individual contribution of HERG1 isoforms, their clinical significance during disease progression and also the underlying regulatory mechanisms. Analysis of HERG1A and HERG1B expression using real-time RT-PCR consistently showed that HERG1A is the predominant isoform in ten HNSCC-derived cell lines tested. HERG2 and HERG3 were also detected. Immunohistochemical analysis of HERG1A expression on 133 HNSCC specimens demonstrated that HERG1A expression increased during tumour progression and correlated significantly with reduced disease-specific survival. Furthermore, our study provides original evidence supporting the involvement of histone acetylation (i.e. H3Ac and H4K16Ac activating marks) in the regulation of HERG1 expression in HNSCC. Interestingly, this mechanism was also found to regulate the expression of another oncogenic channel (Kv3.4) as well as HERG2 and HERG3. These data demonstrate that HERG1A is the predominant and disease-relevant isoform in HNSCC progression, while histone acetylation emerges as an important regulatory mechanism underlying Kv gene expression.

Head and neck squamous cell carcinoma (HNSCC) represents the sixth leading cancer by incidence worldwide. Despite major advancements in cancer diagnosis and treatment, the survival rate for patients with HNSCC has only marginally improved over the past few decades, owing to differences in the biological behaviour of tumours and inadequacies of the present staging system^1^.

Recent advances in genomic and basic research have increased our understanding of the molecular processes governing tumour formation and progression. HNSCC is a heterogeneous disease involving deregulation of multiple pathways linked to cellular differentiation, cell cycle control, apoptosis, angiogenesis, and metastasis^2^. Thus, much work is focused on the identification of better biologic and molecular factors that may serve as prognostic and predictive markers, as well as new targets for therapy^1^,^2^.

Numerous studies have demonstrated the involvement of ion channels in the pathogenesis of various diseases, including cancer^3^,^4^,^5^. Potassium channels were originally identified in excitable cells but they are present in virtually all types of cells, where they are involved in a multitude of physiological functions^5^,^6^,^7^. Because of their oncogenic properties, distribution, modulation and pharmacology, members of the ether à-go-go (EAG) potassium channels family have gained interest as research tools for detection and therapy of different cancers^5^,^8^,^9^,^10^.

We have recently reported that the human ether-à-go-go-related gene 1 (HERG1) potassium channel is frequently aberrantly expressed in an overwhelming high percentage of primary tumours and HNSCC-derived cell lines (>80%), whereas expression was negligible in the corresponding normal epithelia^11^. Furthermore, aberrant HERG1 expression represents a clinically and biologically relevant feature during disease progression and a promising candidate as tumour marker and membrane therapeutic target for HNSCC treatment. Nevertheless, despite HERG1 expression has been extensively studied in numerous cancers, the mechanisms underlying its frequent aberrant expression in tumours remain largely unknown.

Growing evidence indicates that tumours tend to express splice variants or alternative transcripts of channel-encoding genes. Thus, it has been described that various tumours, such as neuroblastoma, small lung carcinoma and leukaemia cell lines^12^,^13^ express an N-terminally truncated splice variant of HERG1, referred to as HERG1B, along with the full-length HERG1 (also termed HERG1A) to form heterotetrameric channels, which show faster deactivation kinetics than those observed for HERG1^14^. In the human heart, although HERG1B is expressed, HERG1A is the predominant isoform, while HERG1B transcript often predominates in tumour cells where the two isoforms coexist^13^.

This work evaluates for the first time the expression of HERG1A and HERG1B isoforms in HNSCC-derived cell lines, their clinical significance during disease progression and the underlying regulatory mechanisms of HERG1 expression in HNSCC, providing original evidence for the involvement of epigenetic regulatory mechanisms, specifically histone acetylation. In addition, the expression of HERG2, HERG3 and other potentially oncogenic Kv channels such as Kv1.3, KCa3.1 and Kv3.4 was also assessed in a panel of HNSCC-derived cell lines, as well as the contribution of histone acetylation to the regulation of aberrant Kv gene expression in HNSCC.

## Results

### Expression of HERG1 isoforms in HNSCC-derived cell lines

**Figure 1:**
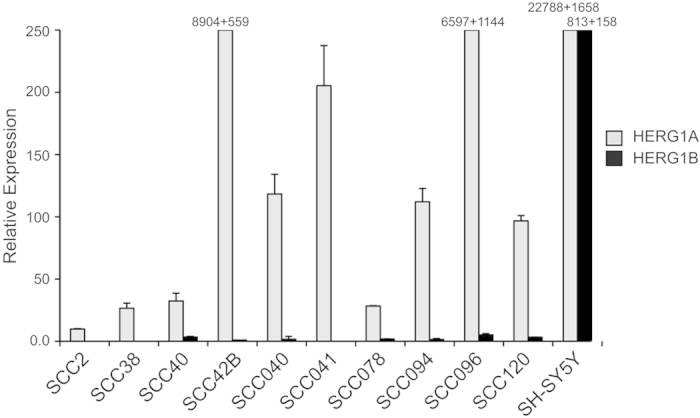
Analysis of HERG1A and HERG1B mRNA levels by real-time RT-PCR in HNSCC-derived cell lines. Data are expressed as the fold change in HERG1A or HERG1B levels in the HNSCC-derived cell lines normalised to L19 mRNA levels. The graph represents the means + SD of two independent experiments performed in quadruplicate. SH-SY5Y cells were included as a positive control.

We first assessed the expression of HERG1A and HERG1B isoforms by real-time RT-PCR in a panel of 10 HNSCC-derived cell lines. HERG1A mRNA expression (corresponding to the full-length isoform) was detected at varying levels in all HNSCC cells (Fig. 1). HERG1B mRNA expression, however, was undetectable in most HNSCC cell lines or detected at very high Ct values (>36). The human neuroblastoma cell line SH-SY5Y, which expresses high levels of both HERG1A and HERG1B isoforms^15^, was used as a positive control. Therefore HERG1A was consistently found to be the predominant isoform in all 10 HNSCC-derived cell lines tested.

In addition, HERG2 and HERG3 were also detected in HNSCC cell lines, although mRNA levels were in general low and quite variable compared to SH-SY5Y cells (Supplementary Figure S1).

### Expression and clinical relevance of HERG1A isoform in HNSCC tissue specimens

**Figure 2:**
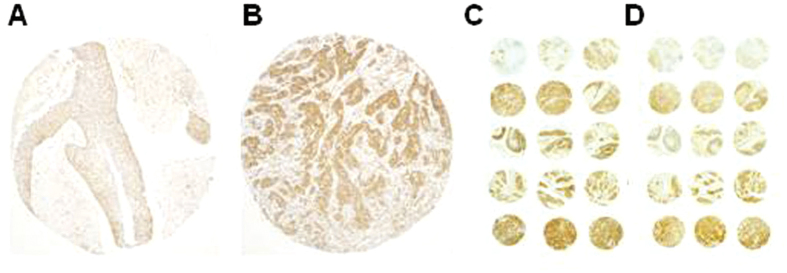
Immunohistochemical analysis of HERG1A expression in HNSCC tissue specimens. Representative examples of head and neck carcinomas showing HERG1A-negative staining (**A**) and strong positive cytoplasmic staining (**B**), five representative cases (*rows* 1 to 5) from a HNSCC tissue microarray stained with either HERG1A-specific (**C**) or HERG1 (pan) antibodies (**D**). Three tissue cores from the same tumour block are shown on each row. A HERG1A-negative case (*row* 1) and four HERG1A-positive cases (*row* 2–5).

Immunohistochemical analysis of HERG1A expression was performed using an isoform-specific antibody on HNSCC tissue microarrays from 133 laryngeal/hypopharyngeal squamous cell carcinomas. Immunostaining was successfully evaluated in 125 (94%) of 133 cases. 113 (90%) of the 125 tumours exhibited HERG1A-positive expression (moderate to strong expression, Fig. 2A,B), preferentially detected in the cytoplasm. HERG1A expression was negligible in both normal epithelium and stromal cells. A highly significant correlation was found between HERG1A staining (Fig. 2C) and that previously evaluated using anti-HERG1 (pan) antibody shown in Fig. 2D (Supplementary Table S1; n = 123; *P* < 0.001; Kendall’s tau correlation test).

In good agreement to our previous observations using the anti-HERG1 pan (CT) antibody^11^, using the same cohort of HNSCC patients we found that the expression of HERG1A isoform (specifically obtained with an anti-HERG1A (NT) antibody) also increased during tumour progression and associated significantly with lymph node metastasis (N1-3 *versus* N0, *P* = 0.018, Supplementary Table S2), advanced disease stages (III and IV *versus* I-II, *P* < 0.001), with tumour recurrence (*P* = 0.028) and distant metastasis (*P* = 0.038).

**Figure 3:**
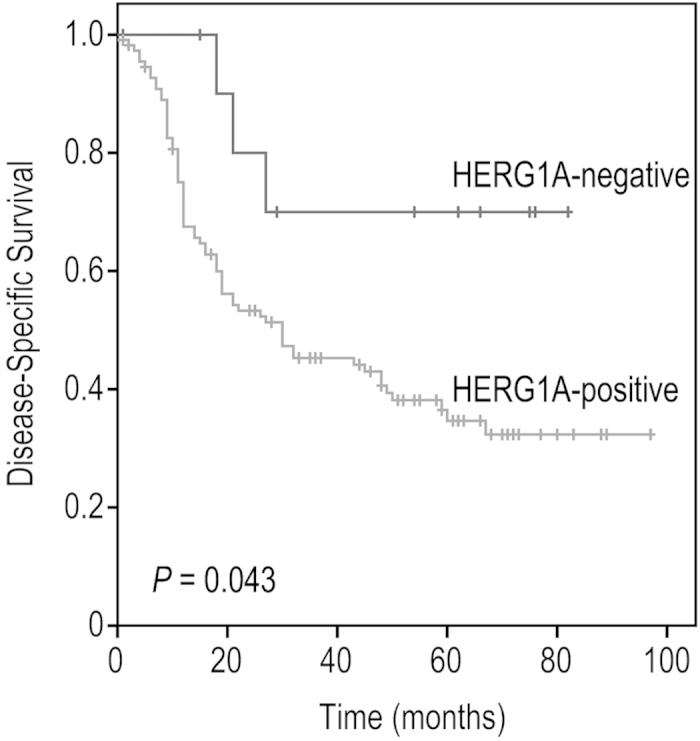
Kaplan-Meier disease-specific survival curves in patients with HNSCC categorised by HERG1A protein expression (positive *versus* negative). *P* values were estimated using the log-rank test. *P* ≤ 0.05 was considered statistically significant.

The median follow-up of the whole series was 24.5 months (range, 1–97 months), and the median follow-up of the patients alive at the last visit was 67.5 months (range, 48–97 months). 15 patients died for other causes not related to the primary tumour before 36 months of follow-up. These cases were excluded from the recurrence analysis. Univariate Kaplan-Meier analysis showed a significant correlation between HERG1A-positive expression and reduced disease-specific survival (log-rank test, *P* = 0.043, Fig. 3). Other variables associated with a poorer prognosis were hypopharyngeal location of the tumour (HR = 2.13; CI 95% 1.3–3.5; *P* = 0.002), presence of nodal metastases (HR = 3.71; CI 95% 1.9–7.1; *P* < 0.001), and poor histological differentiation (HR = 1.5; CI 95% 1.1–2.1; *P* = 0.012). In multivariate analysis the only parameters independently associated with a reduced disease-specific survival were the presence of nodal metastasis (HR = 3.5; CI 95% 1.8–6.7; *P* < 0.001) and poor histological differentiation (HR = 1.4; CI 95% 1.05–1.9; *P* = 0.047).

### Contribution of Epigenetic Regulatory Mechanisms to HERG1 Expression in HNSCC

**Figure 4:**
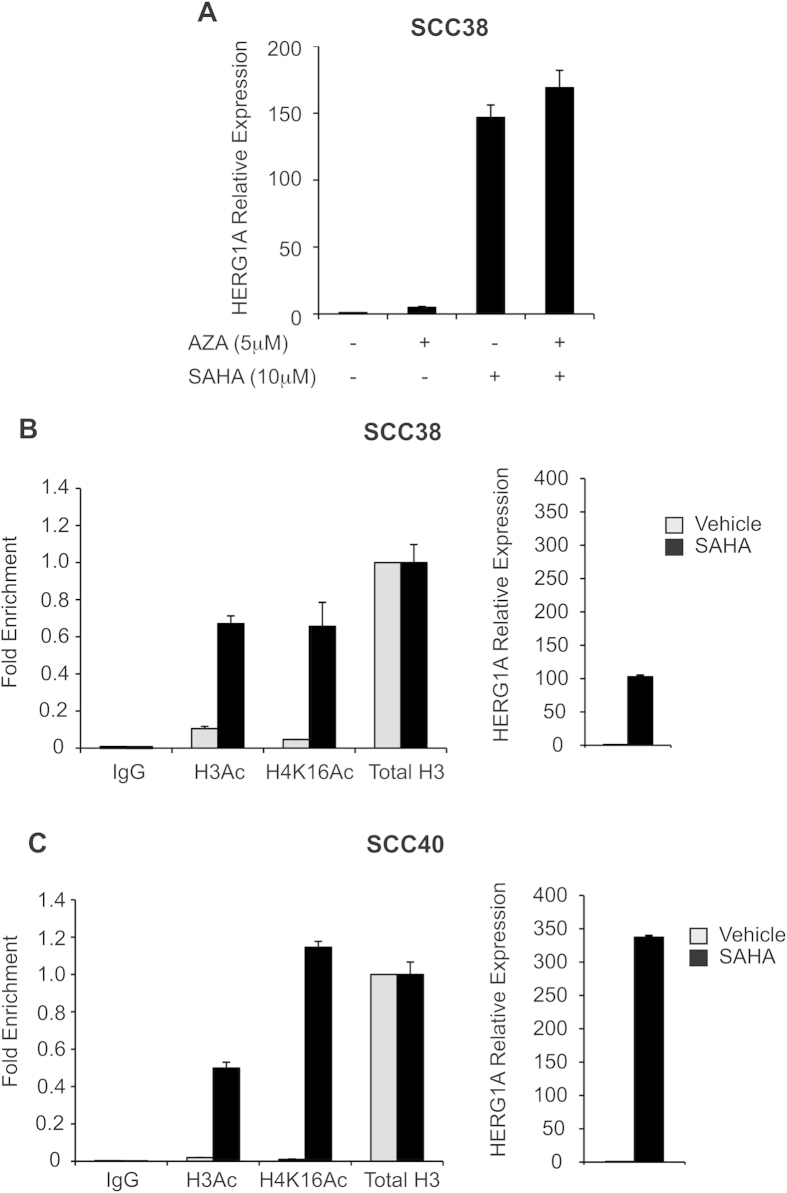
Epigenetic regulation of HERG1A expression. (**A**) HERG1A mRNA expression levels in SCC38 cells treated with either vehicle, 5 μM AZA for 96 h, 10 μM SAHA for 24 h or a combination of both chemical agents. HERG1A expression was analysed by real-time RT-PCR and data were normalised to RPL19 mRNA levels. Histone H3 and H4 acetylation status measured by qChIP in either SCC38 cells (**B**) or SCC40 cells (**C**) treated with vehicle or 10 μM SAHA for 24 h. *In vivo* cross-linked chromatin was immunoprecipitated with either anti-acetyl-histone H3 (H3Ac), anti-acetyl-histone H4 (Lys 16) (H4K16Ac), histone H3 (total H3), or rabbit control IgG (IgG) and the relative histone acetylation levels analysed by qPCR. Data were normalised to IgG and relative to H3 immunoprecipitated samples. *The right panel* shows HERG1A expression analysis by real-time RT-PCR in either SCC38 cells (**B**) or SCC40 cells (**C**) treated with vehicle or 10 μM SAHA for 24 h.

To determine whether HERG1 expression could be regulated by epigenetic mechanisms, we treated HNSCC cells with either demethylating agent 5-aza-2′-deoxycytidine (AZA), the histone deacetylase inhibitor suberoylanilide hydroxamic acid (SAHA) or combinations. We found that HERG1A mRNA levels greatly increased upon SAHA treatment in SCC38 cells, whereas the demethylating agent AZA had only a marginal effect on HERG1A expression (Fig. 4A).

To study the possible contribution of histone post-translational modification to the aberrant expression of HERG1 in HNSCC, two different HNSCC-cell lines (SCC38 and SCC40) were treated with the histone deacetylase inhibitor SAHA and histone acetylation status at HERG1 promoter was analysed by ChIP experiments using antibodies against both histone H3 acetylation (H3Ac) and histone H4 lysine 16 acetylation (H4K16Ac). Results consistently showed an enrichment of both histone activating marks at the HERG1 promoter upon SAHA treatment that parallels increased HERG1A mRNA expression in both HNSCC-derived cell lines (Fig. 4B,C), suggesting the contribution of histone acetylation to the regulation of aberrant HERG1 expression in HNSCC.

Given that, in addition to HERG1A, HERG2 and HERG3 mRNAs were also detected in HNSCC cells, the role of histone acetylation as a potential transcriptional regulatory mechanism was investigated. Furthermore, we extended this study to determine whether this mechanism could also apply to other potassium channels, such as Kv1.3, KCa3.1 and Kv3.4, previously proven to have oncogenic properties^16^,^17^,^18^,^19^.

**Figure 5:**
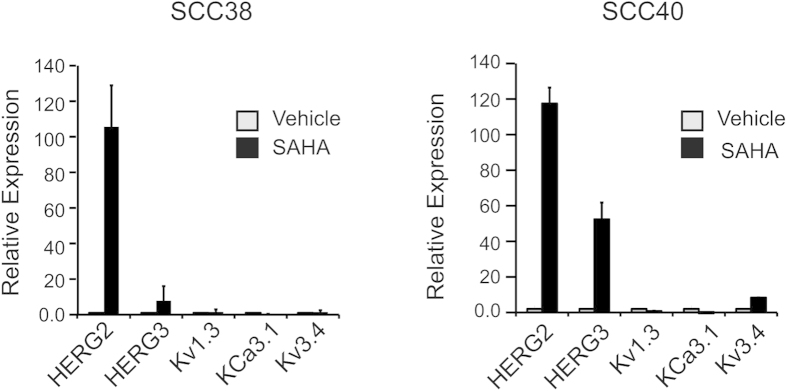
Regulation of Kv expression by histone acetylation. mRNA expression levels of HERG2, HERG3, Kv1.3, KCa3.1 and Kv3.4 in SCC38 cells and SCC40 cells treated with either vehicle or 10 μM SAHA for 24h. mRNA expression was analysed by real-time RT-PCR and data normalised to RPL19 mRNA levels.

Thus, we found that the expression of HERG2 and HERG3 was highly induced upon SAHA treatment in both SCC38 and SCC40 cells, whereas Kv3.4 mRNA levels were only slightly increased in SCC40 cells. In contrast, expression of Kv1.3 and KCa3.1 was very low in HNSCC cells and unaffected by SAHA (Fig. 5A,B).

**Figure 6:**
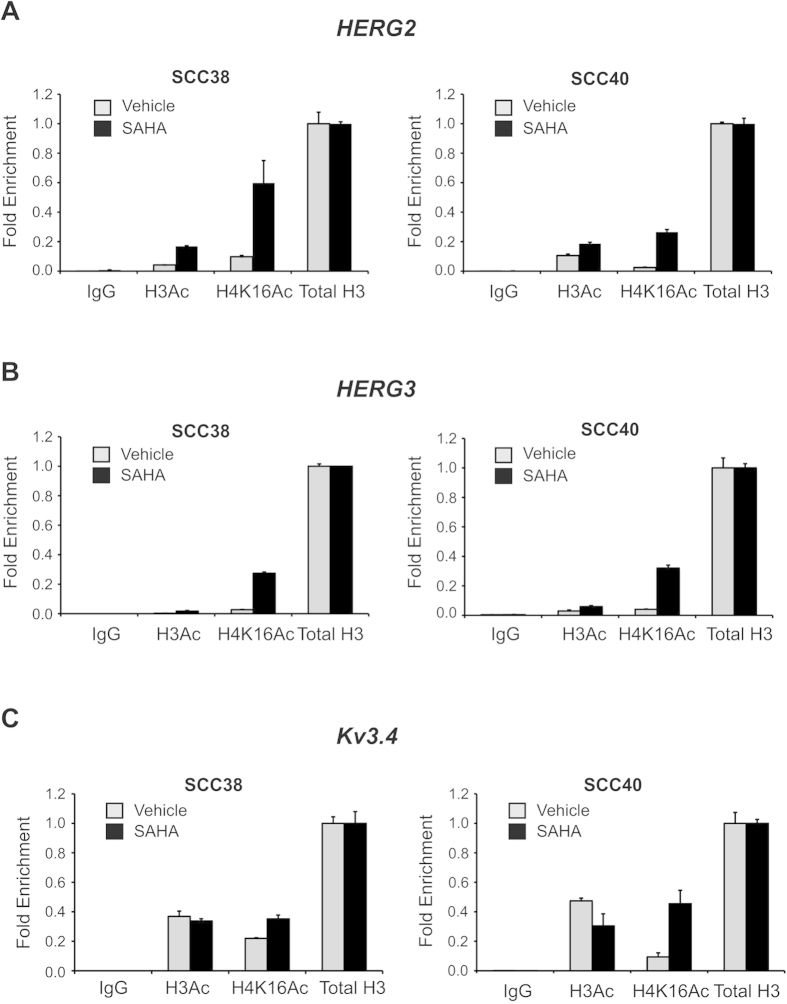
Histone H3 and H4 acetylation status of *HERG2* (**A**), *HERG3* (B) or *Kv3.4* (**C**) measured by qChIP in either SCC38 cells or SCC40 cells treated with vehicle or 10 μM SAHA for 24 h. *In vivo* cross-linked chromatin was immunoprecipitated with either anti-acetyl-histone H3 (H3Ac), anti-acetyl-histone H4 (Lys 16) (H4K16Ac), histone H3 (total H3), or rabbit control IgG (IgG) and the relative histone acetylation levels analysed by qPCR. Data were normalised to IgG and relative to H3 immunoprecipitated samples.

Using ChIP assays, we further demonstrated that enrichment of histone activating marks (in particular, H4K16Ac) occurred at each promoter upon SAHA treatment in SCC38 and SCC40 cells (Fig. 6A–C).

## Discussion

It has been increasingly documented that ion channels, and in particular potassium channels, are implicated in all steps of carcinogenesis and tumour progression^3^,^5^. The contribution of potassium channels to the neoplastic phenotype involves several hallmarks of cancer such as regulation of cell proliferation, resistance to apoptosis, tumour angiogenesis, invasiveness and metastatic spread^6^,^7^,^8^,^9^,^10^,^11^. The oncogenic properties of potassium channels together with their extracellular accessibility and functional modulation make them excellent targets for pharmacological and therapeutic interventions^5^,^9^,^20^.

We have recently demonstrated that HERG1 potassium channel is frequently aberrantly expressed in an extraordinarily high percentage of primary tumours (87%) and HNSCC-derived cell lines (100%)^11^. HERG1 expression was also detected in a considerable proportion of laryngeal dysplasias (41%). More importantly, HERG1 expression significantly correlated with increased laryngeal cancer risk, uncovering its potential clinical utility as a novel biomarker for cancer risk assessment^11^.

The present study further extends these data to demonstrate that HERG1A is the predominant isoform in HNSCC progression, even though it has been considered that HERG1B isoform is predominant in tumour cells^8^,^13^. Thus, analysis of HERG1A and HERG1B expression by using real-time RT-PCR consistently showed that HERG1A isoform is the most abundant in all ten HNSCC-derived cell lines tested. In addition, HERG1A expression was also frequently detected in HNSCC specimens (90%) and significantly correlated with lymph node metastasis, advanced disease stages, tumour recurrence, distant metastasis, and reduced disease-specific survival. These results reinforce that HERG1A is the predominant and disease-relevant isoform in HNSCC progression. Hence, consistent with our *in vivo* findings, aberrant HERG1 expression may confer a proliferative advantage to the cells carrying this alteration and the acquisition of invasive potential, thus favouring (*i*) progression of precancerous lesions to invasive carcinoma in early stages of tumourigenesis, and (*ii*) the acquisition of a truly invasive and metastatic potential in late stages during HNSCC progression, facilitating tumours to grow faster and with increased aggressiveness, which ultimately leads to disease progression.

HERG1 has similarly been found frequently aberrantly expressed in other types of cancer^13^,^21^,^22^,^23^,^24^, where HERG1 expression commonly correlated with tumour aggressiveness, poor prognosis and metastatic disease. Nevertheless, most studies do not evaluate the individual contribution and clinical significance of HERG1 isoforms and little is known about the molecular mechanisms underlying their frequent aberrant expression in human cancers. This study investigates for the first time the possible contribution of epigenetic transcriptional regulatory mechanisms. Since we observed that HERG1A mRNA levels in HNSCC cells specifically increased by treatment with the histone deacetylase inhibitor SAHA, we further assessed the histone acetylation status at the HERG1 promoter using ChIP experiments. Our results provide original evidence showing the involvement of histone acetylation in the regulation of HERG1 expression in HNSCC. In particular, enrichment of histone activating marks such as H3Ac and H4K16Ac was significantly associated to HERG1 expression in HNSCC-derived cell lines. Interestingly, we also provide novel findings demonstrating that this mechanism may contribute to the aberrant expression of a number of EAG family members (HERG1, HERG2, HERG3 and hEAG1^10^) as well as other potentially oncogenic Kv channels such as Kv3.4^19^. Therefore, according to these data histone acetylation emerges as an important regulatory mechanism underlying aberrant Kv expression in HNSCC, and presumably in other cancers. Noteworthy HDAC inhibitors (HDACi) such as SAHA (Vorinostat) are currently being evaluated in clinical trials for cancer therapy^25^. According to our data, caution must nonetheless be taken when using these pleiotropic agents with widespread effects, as in addition to derepressing TSGs silenced in cancer, HDACi could activate and promote HERG1 and hEAG1 oncogenic potential.

Despite the primary function of voltage-dependent ion channels is to regulate potassium flux through the cell membrane, recent studies have shown that HERG1 channels may exert pleiotropic effects in cancer cells by triggering and modulating intracellular signalling cascades through the formation of macromolecular complexes with membrane receptors, especially integrins^8^. Based on current evidence, it has been hypothesized that the activity of HERG1 channels inside these complexes modulates the function of the partner proteins mainly via conformational coupling, independently of the ion-conducting function. In addition, it has been proposed that the HERG1-centered plasma membrane complexes, being specific to cancer cells, could represent novel targets for antineoplastic therapy^13^.

In summary, beyond demonstrating for the first time that HERG1A potassium channel subunit is the predominant and disease-relevant isoform associated to head and neck tumour progression and metastatic spread, this study provides novel insights into the underlying regulatory mechanisms of HERG1 expression in HNSCC with original evidence supporting the involvement of histone acetylation. According to our data, HERG1A may favour HNSCC progression and metastasis through an enhancement of cell proliferation and invasiveness, postulating this potassium channel subunit as a useful antiproliferative and antimetastatic target for cancer therapy. Nevertheless, to fully establish the tumour-specific isoform and also key molecular and biophysical features of HERG1 deregulation in the different cancers would be fundamental as it will undoubtedly open new avenues for therapeutic strategies to target specifically and effectively HERG1 oncogenic properties.

## Methods

### Patients and tissue specimens

Surgical tissue specimens from 133 patients with laryngeal or hypopharyngeal squamous cell carcinoma who underwent surgical treatment at the Hospital Universitario Central de Asturias between 1996 and 2005 were retrospectively collected, in accordance to approved institutional review board guidelines. All experimental protocols were approved by the Institutional Ethics Committee of the Hospital Universitario Central de Asturias and by the Regional CEIC from Principado de Asturias (date of approval 18th of July 2013; approval number: 81/2013) for the project PI13/00259. Written informed consent was obtained from all patients. Representative tissue sections were obtained from archival, paraffin-embedded blocks and the histological diagnosis was confirmed by an experienced pathologist. Three morphologically representative areas were selected from each individual tumour block to construct 5 tissue microarray blocks, as described previously^11^. In addition, each tissue microarray also contained three cores of normal epithelium as an internal control.

All patients had a single primary tumour, microscopically clear surgical margins and received no treatment prior to surgery. A high percentage of patients who developed distant metastasis was included (52 of 133, 39%) to investigate the possible role of HERG1 expression in metastatic dissemination. Only five patients were women, the mean age was 60 years (range 38 to 86 years). All but three patients were habitual tobacco and alcohol consumers. 71 (53%) of 133 patients received postoperative radiotherapy. The characteristics of the patients studied and the clinicopathological features of their tumours (site, pT classification, pN classification, disease stage, and histopathologic grade) are shown in Supplementary Table S2. The stage of disease was determined after the surgical resection of the tumour according to the TNM system of the International Union against Cancer (6th Edition). The histological grade was determined according to the degree of differentiation of the tumour (Broders’ classification).

### Immunohistochemistry

The formalin-fixed, paraffin-embedded tissues were cut into 3-μm sections and dried on Flex IHC microscope slides (Dako). The sections were deparaffinized with standard xylene and hydrated through graded alcohols into water. Antigen retrieval was performed using Envision Flex Target Retrieval solution, high pH (Dako). Staining was done at room temperature on an automatic staining workstation (Dako Autostainer Plus) with rabbit polyclonal anti-HERG1A (NT) antibody (Enzo Life Sciences, Inc.) at 1:250 dilution using the Dako EnVision Flex + Visualization System (Dako Autostainer). Counterstaining with haematoxylin was the final step.

Since HERG1A staining showed a homogeneous distribution, a semiquantitative scoring system based on staining intensity was applied. Immunostaining was scored blinded to clinical data by two independent observers as negative (0), weakly (1+), moderately (2+) or strongly positive (3+). Scores ≥2 were considered as HERG1A-positive expression.

### Cell culture

The HNSCC-derived cell lines SCC2, SCC38, SCC40 and SCC42B were kindly provided by Dr. R. Grenman (Department of Otolaryngology, University Central Hospital, Turku, Finland)^26^ and SCC040, SCC041, SCC078, SCC094, SCC096A and SCC120 by Dr. MA Hermsen^27^. Cells were grown in DMEM supplemented with 10% foetal bovine serum, 100 units/mL penicillin, 200 μg/mL streptomycin, 2mmol/L L-glutamine, 20 mmol/L HEPES (pH 7.3), and 100 μmol/L non-essential amino acids.

### Real-time RT-PCR

Total RNA was extracted using Trizol reagent (Invitrogen Life Technologies), and cDNA synthesized with Superscript II RT-PCR System (Invitrogen Life Technologies), according to manufacturer’s protocols. Gene expression was analysed by real-time PCR using the ABI PRISM 7500 Sequence detector following Applied Biosystems’ SYBR Green Master Mix protocol. Reactions were carried out using primers specific for HERG1A (matching exons 5 and 6), primers specific for HERG1B (matching exons 1 and 2, described in^13^) and for the constitutively expressed L19 ribosomal coding gene as internal control. Samples were analysed in triplicates and template-free blanks were also included. Primer sequences are summarised in Supplementary Table S3. The relative mRNA expression was calculated using the 2^−ΔΔ*C*T^ method.

### Treatment of HNSCC-derived cells with epigenetic agents

To determine the effect of the demethylating agent 5-aza-2′-deoxycytidine (AZA) (Sigma-Aldrich, Madrid, Spain) and the histone deacetylase inhibitor suberoylanilide hydroxamic acid (SAHA) (Sigma-Aldrich, Madrid, Spain) on Kv gene expression, HNSCC cells were treated with either 5 μM AZA for 96 h, 10 μM SAHA for 24 h, or with a combination of both chemical agents. Growth medium with the corresponding treatment was replaced every 48h. After treatment, cells were harvested and processed for real-time RT-PCR and ChIP assays.

### Chromatin immunoprecipitation assays and PCR quantification (qChIP)

All the procedure was performed on ice, with buffers containing protease inhibitors (cOmplete EDTA-free, Roche Diagnostics, Indianapolis, IN). Briefly, HNSCC cells either untreated or treated for 24h with 10 μM SAHA, were incubated with 1% formaldehyde for 30 min in a cold room with constant agitation. Chromatin cross-linking was stopped by the addition of 125 mM glycine, and cells were washed in cold phosphate-buffered saline (PBS). Subsequent proceedings were performed as previously described^10^. Chromatin was sheared with a Bioruptor (Diagenode, Liège, Belgium) to an average length of 300 – 600 bp. The following polyclonal antibodies were used: anti-acetyl-histone H3 (H3Ac; Millipore, # 06–599), anti-acetyl-histone H4 (Lys 16) (H4AcK16; Millipore, # 07–329), histone H3 – ChIP grade (H3; Abcam, # ab1791), rabbit control IgG – ChIP grade (IgG; Abcam, # ab46540). The quality of the antibodies used for acetyl-histone H4 and H3 has been previously proved by Western blot analysis^10^. Quantitative analysis of *HERG1* promoter region (−68, +23) was performed by real-time PCR using Taqman Universal PCR Master Mix on an ABI PRISM 7500 Sequence Detection System (Applied Biosystems, Foster City, CA). Quantitative analyses of *HERG2, HERG3* and *Kv3.4* gene promoters were carried out by real-time PCR using Power SYBR Green on an Applied Biosystems StepOnePlus™ Real-Time PCR System. Primers and probe sequences are shown in Supplementary Table S3. Quantitative real-time PCR data were normalised *versus* total IgG and total H3 immunoprecipitated values.

### Statistical analyses

The χ^2^ test and Fisher’s exact test were used for comparison between categorical variables. The correlation between HERG1A and HERG (pan) protein expression was estimated by Kendall’s tau correlation test. For time-to-event analysis, Kaplan-Meier curves were plotted. Differences between survival times were analysed by the log-rank method. Cox proportional hazards models were utilised for univariate and multivariate analyses. The HR with 95% CI and *P* values were reported. All tests were two-sided. The values of *P* ≤ 0.05 were considered statistically significant.

## Additional Information

**How to cite this article**: Menéndez, S.T. *et al*. HERG1A potassium channel is the predominant isoform in head and neck squamous cell carcinomas: evidence for regulation by epigenetic mechanisms. *Sci. Rep.* **6**, 19666; doi: 10.1038/srep19666 (2016).

## Supplementary Material

Supplementary Information
