# Supplementary material for: HERG1A potassium channel is the predominant isoform in head and neck squamous cell carcinomas: evidence for regulation by epigenetic mechanisms
Source: Sci Rep. 2016 Jan 20;6:19666. doi: 10.1038/srep19666 (PMC4726400; doi:10.1038/srep19666)
Supplement: Supplementary Information [file srep19666-s1.pdf]

SUPPLEMENTARY INFORMATION

Title: HERG1A potassium channel is the predominant isoform in head and neck squamous cell carcinomas: evidence for regulation by epigenetic mechanisms

AUTHORS:

Sofía Tirados Menéndez[1]‡, María Ángeles Villaronga[1]‡, Juan P. Rodrigo[1], Saúl Álvarez-Teijeiro[1], Rocío G. Urdinguio[2], Mario F. Fraga[2,3], Carlos Suárez[1], Juana María García-Pedrero[1].

[1] Servicio de Otorrinolaringología, Hospital Universitario Central de Asturias and Instituto Universitario de Oncología del Principado de Asturias, Oviedo, Spain.

[2] Unidad de Epigenética del Cáncer, Instituto Universitario de Oncología del Principado de Asturias, Universidad de Oviedo, Spain.

[3] Department of Immunology and Oncology, National Center for Biotechnology, CNB-CSIC, Cantoblanco, Madrid E-28049, Spain

‡These authors contributed equally to this work.

* ***Correspondence***:

Juana María García-Pedrero, PhD

Hospital Universitario Central de Asturias

Laboratorio 2 ORL - IUOPA

33006 Oviedo, Spain

Phone: +34 985107937

E-mail: juanagp@ficyt.es

Table S1. Correlation between HERG1A (NT) and HERG1 pan (CT) immunostaining (N=123)

Characteristic	No.	HERG1A protein expression	
		Negative	Positive
**HERG1 protein expression**			
Negative	15	8	7
Positive	108	3	105

† Kendall's Tau = 0.580; *P* <0.001

Table S2. Associations between HERG1A protein expression and clinicopathological findings, site, relapse and disease outcome

Characteristic	No.	HERG1A protein expression (%)	*P*
**- pT classification**			
T1-T2	35	29 (83)	0.203[#]
T3	45	42 (93)	
T4	45	42 (93)	
**- pN classification**			
N0	40	32 (80)	0.018[†]
N1-3	85	81 (95)	
**- Disease stage**			
I-II	15	9 (60)	<0.001[#]
III	24	22 (92)	
IV	86	82 (95)	
**- Pathological grade**			
Well differentiated	35	28 (80)	0.048[#]
Moderately differentiated	56	53 (95)	
Poorly differentiated	34	32 (94)	
**- Site**			
Pharynx	61	58 (95)	0.128[†]
Larynx	64	55 (86)	
**- Tumour Recurrence**‡			
No	35	29 (80)	0.028[†]
Yes	75	72 (96)	
**- Distant Metastasis**			
No	60	52 (87)	0.038[†]
Yes	50	49 (98)	
**- Disease status**			
Alive without disease	29	24 (83)	0.045[†]
Dead of index cancer	70	67 (96)	
Died of other causes	14	13 (85)	
**Total Cases**	125	113 (90)	

[#] Chi-square and [†] Fisher's exact tests.
‡ 15 patients who died from causes not related to the index tumour before 36 months of follow-up were excluded from the recurrence analysis.

Table S3. Primers used in real-time RT-PCR and qChIP *

GENE	FORWARD (5’ -> 3’)	REVERSE (5’ -> 3’)
HERG1A	ACCGTGAGATCATAGCACCTAA	ACAGGACCTGGGTGACCTTCT
HERG1B #	ACAGGACCTGGGTGACCTTCT	ACGTCGGCGCCCAGGGACA
HERG2	GGCCTTGGTTCCTATAGCCT	TCCAGGGGATGTAGAGGTGA
HERG3	CGGACCTCTTGACCATTCCT	ATTGTCTTCGCTGGCATGTC
Kv 1.3	GCCAGTTCCCGTGATTGTTT	CGTTTTGAAAGGGGTCTGGG
KCa 3.1	GTTCGTGGCCAAGCTTTACA	GCATTAACAGCCTGCCTCTC
Kv3.4	CGCTCTTCGAGGATCCCTACT	TGGTGATGGAGACCAGGATGA
HERG2 (KCNH6)*	ACGCTGCTGAGCCCG	CCTGCCCGGAGCCACA
HERG3 (KCNH7)*	GCGGGTGTCTAGAAGTGGTG	CTCTTTGTGGCAGAGCATCC
Kv 1.3 (KCNA3)*	GCAGCCTGACTCCCCGT	GCCCTCATGTTGTACCTCGG
KCa 3.1 (KCNN4)*	TGTTTGGGTGTGGCTCTTTG	GTCCACACACAGGCACAAAT
Kv 3.4 (KCNC4)*	TGCCTCCTCTTCGTCTCCTC	CTCCAAACACCCTTCCCGAG
HERG1 (KCNH2)*	CCCGCAGTCCAGTCTTGG	CAGCACTAGGCTTCGGGTG
	**Probe** 6-FAM-CGCCCGTGCTCGGCTTGG-TAMRA	

Primer sequences taken from Pillozi *et al.* [13].

Figure Legends

Figure S1. Analysis of HERG2 and HERG3 mRNA levels by real-time RT-PCR in HNSCC-derived cell lines. Data are expressed as the fold change in HERG2 or HERG3 levels in the HNSCC-derived cell lines normalised to L19 mRNA levels. The graphs represent the means + SD of at least two independent experiments performed in quadruplicate. SH-SY5Y cells served as a positive control.

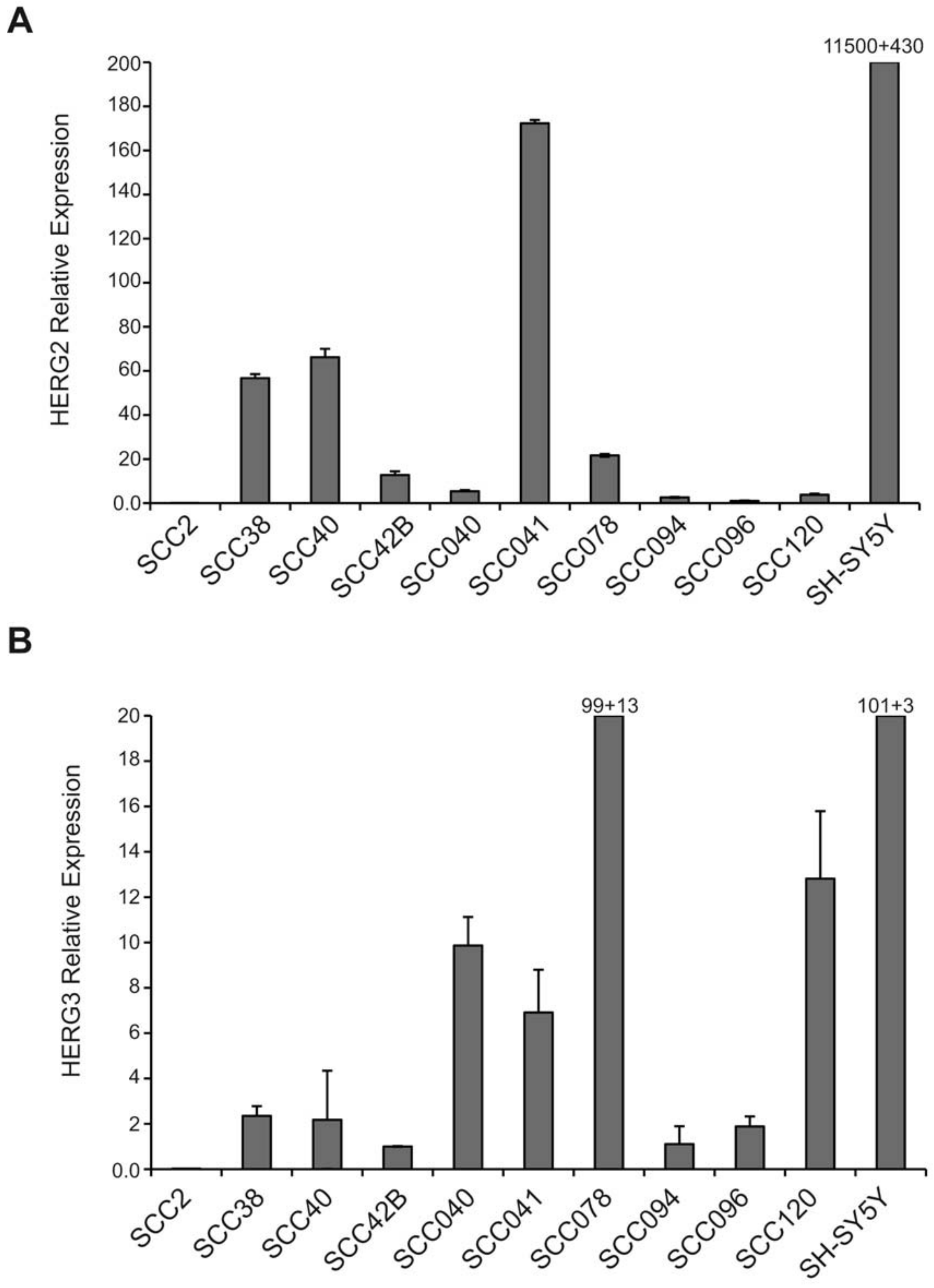
A
HERG2 Relative Expression
0.0
20
40
60
80
100
120
140
160
180
200
11500+430
SCC2
SCC38
SCC40
SCC42B
SCC040
SCC041
SCC078
SCC094
SCC096
SCC120
SH-SY5Y
B
HERG3 Relative Expression
0.0
2
4
6
8
10
12
14
16
18
20
99+13
101+3
SCC2
SCC38
SCC40
SCC42B
SCC040
SCC041
SCC078
SCC094
SCC096
SCC120
SH-SY5Y
